# Modification of Preservative Fluids with Antioxidants in Terms of Their Efficacy in Liver Protection before Transplantation

**DOI:** 10.3390/ijms25031850

**Published:** 2024-02-03

**Authors:** Aneta Ostróżka-Cieślik

**Affiliations:** Department of Pharmaceutical Technology, Faculty of Pharmaceutical Sciences in Sosnowiec, Medical University of Silesia, Kasztanowa 3, 41-200 Sosnowiec, Poland; aostrozka@sum.edu.pl

**Keywords:** liver transplantation, antioxidants, organ preservation solution

## Abstract

Transplantation is currently the only effective treatment for patients with end-stage liver failure. In recent years, many advanced studies have been conducted to improve the efficiency of organ preservation techniques. Modifying the composition of the preservation fluids currently used may improve graft function and increase the likelihood of transplantation success. The modified fluid is expected to extend the period of safe liver storage in the peri-transplantation period and to increase the pool of organs for transplantation with livers from marginal donors. This paper provides a literature review of the effects of antioxidants on the efficacy of liver preservation fluids. Medline (PubMed), Scopus, and Cochrane Library databases were searched using a combination of MeSH terms: “liver preservation”, “transplantation”, “preservation solution”, “antioxidant”, “cold storage”, “mechanical perfusion”, “oxidative stress”, “ischemia-reperfusion injury”. Studies published up to December 2023 were included in the analysis, with a focus on publications from the last 30 years. A total of 45 studies met the inclusion criteria. The chemical compounds analyzed showed mostly bioprotective effects on hepatocytes, including but not limited to multifactorial antioxidant and free radical protective effects. It should be noted that most of the information cited is from reports of studies conducted in animal models, most of them in rodents.

## 1. Introduction

Liver injury due to ischemia and reperfusion is a significant problem in the peri-transplantation period. Warm ischemia results in damage to hepatocytes through the activation of Kupffer cells and pro-inflammatory cytokines. Cold IRI (ischemia–reperfusion injury) results in the dysfunction of hepatic sinusoidal endothelial cells and impaired microcirculation [1,2,3,4]. Endothelin-1 increase and nitric oxide decrease cause vasoconstriction [5]. Oxidative phosphorylation in mitochondria is inhibited. ATP stores are depleted and, consequently, the activity of the cell’s active transport system and membrane potential decreases. The level of Ca^2+^ ions in the cell increases, which affects the activation of cell-damaging enzymes such as phospholipases, endonucleases, proteases, and ATP-ase. The rate of anaerobic glycolysis increases. Mitochondria become swollen and highly permeable channels are formed in their inner membrane. Cytochrome c is released into the cytoplasm, which can direct the cell into the apoptosis pathway. The cell membrane and elements of the cytoskeleton are damaged [3,6].

Oxidative stress generates the production of ATP metabolites, accompanied by a sharp increase in the production of superoxide anion radical (O_2_^•−^), hydrogen peroxide (H_2_O_2_), and hydroxyl radicals (-OH). These initiate circulatory disturbances and a cascade of inflammatory reactions. Mitochondrial membrane morphology and permeability are altered. Damage to the mitochondrial respiratory chain leads to inhibition of oxidative phosphorylation and disruption of energy metabolism. Free oxygen radicals formed in mitochondria cause damage to DNA and organelle proteins and peroxidation of membrane lipids, consequently leading to cell death by apoptosis or necrosis [7,8].

Recent years have seen an increase in research into developing organ perfusion and preservation techniques to minimize ischemia-related graft damage and improve marginal donor utilization rates. The optimization of organ preservation techniques, i.e., static cold storage (SCS; 0–4 °C), hypothermic machine perfusion (HMP, 0–4 °C), subnormothermic machine perfusion (SNMP; 20–30 °C), normothermic machine perfusion (NMP; 32–37 °C), and the introduction of novel substances into preservation fluid compositions, can significantly improve organ function before transplantation. A relatively new generation of organ preservation technology is NMP, which allows blood flow in the organ to be reconstructed and its vital functions to be assessed outside the human body. Transplant rejection rates using this method are 50% lower compared to static cold storage [9]. 

Modifying the composition of the preservation fluids currently used may improve graft function and increase the likelihood of transplantation success. The modified fluid is expected to extend the period of safe liver storage in the peri-transplantation period and to increase the pool of organs for transplantation with livers from marginal donors. This paper provides a literature review of the effects of antioxidants on the efficacy of liver preservation fluids.

## 2. Materials and Methods

### 2.1. Focused Questions

The research question was “Does modifying the composition of preservative fluids with antioxidants affect the efficacy of liver preservation before transplantation?”

### 2.2. Eligibility Criteria

Medline (PubMed), Scopus, and Cochrane Library databases were searched using a combination of MeSH terms: “liver preservation”, “transplantation”, “preservation solution”, “antioxidant”, “cold storage”, “mechanical perfusion”, “oxidative stress”, “ischemia-reperfusion injury”. In addition, reference lists of found publications and bibliographies of studies were searched. Articles containing keywords in the titles or abstracts were included. Articles published in peer-reviewed journals were included in the review after reading the full text. Studies published up to December 2023 were included in the analysis, with a focus on publications from the last 30 years. Inclusion criteria: original articles in English, containing data from basic, preclinical, and clinical studies. Various storage techniques and liver preservation times were included. Exclusion criteria: articles in which the antioxidant was applied in a form other than a component of organ perfusion and preservation fluid (e.g., antioxidant administered by intraperitoneal injection, intravenous injection, bolus through the tail vein, oral delivery, application directly to the organ, modification of blood and its derivatives by the addition of antioxidants). Review articles, letters to the editor, books, studies published on websites and newsletters were not considered. Papers that did not meet the set criteria were not included in the review, thus ensuring the due quality of the literature selected for the review.

## 3. Strategies Based on Modifications of Preservative Solutions with Antioxidants

Transplant fluids provide the environment in which organs are stored during the peri-transplantation period. They extend the ischemic period of the graft and prevent the development of damage during this time. Preserving the optimal vital functions of the organ improves its function in the subsequent postoperative period. The development of an optimal preservative fluid is an important element of transplant success. The substances contained in it should have a multidirectional effect, including anti-inflammatory and cytoprotective effects, which will increase the effectiveness of the transplants performed. Commercially available preservative fluids have been discussed in detail by me in previous publications [10,11,12,13]. Studies indicate that UW (University of Wisconsin), HTK (histidine–tryptophan–ketoglutarate), and IGL-1 (Institut Georges Lopez-1) are the most commonly used in liver transplantation [14]. Antioxidants are important components of organ perfusion and preservation fluids. An analysis of the available literature (Table 1 and Table 2) indicates that the challenge is to develop a preservative fluid formulation for effective liver preservation in which the antioxidant is compatibility with the other ingredients, demonstrates efficacy and safety of use, and does not complicate the manufacturing process.

### 3.1. Enzymatic Antioxidant

Manganese superoxide dismutase (MnSOD) is an enzyme located in the mitochondria and has a protective function against oxidative damage. The anti-inflammatory effect of MnSOD is mainly due to its superoxide anion scavenging ability. Superoxide anions exhibit pro-inflammatory effects by causing DNA damage, peroxynitrite ion formation, lipid peroxidation, and oxidation, and recruitment of neutrophils to sites of inflammation [60]. MnSOD converts superoxide anion radicals to hydrogen peroxide and oxygen in mitochondria [61]. It has been suggested that this has therapeutic effects in liver diseases associated with increased oxidative stress. This is mainly due to the beneficial effects of MnSOD on hepatic endothelial function [62]. Taurine (TAU, 2-aminoethane sulfonic acid) is a sulfuramino acid and a natural antioxidant. It exhibits limited oxygen-free radical scavenging capacity. It is believed that by maintaining an optimal taurine concentration in the mitochondria, ROS production can be controlled. This compound has a stabilizing effect on cell membranes and inhibits enzymes that induce the production of oxygen-free radicals [63]. Its protective effect on liver ischemia is currently disputed [21,64,65,66]. Hide et al. [54] added a recombinant form of the antioxidant human manganese superoxide dismutase to Celsior fluid. rMnSOD prevented oxidative stress and reduced the expression of vWF (von Willebrand factor) and ICAM1 (intracellular adhesion molecule 1). Studies were performed in a model of primary cultured LSECs, rat livers, and human samples. The addition of rMnSOD to the Celsior solution protected rat and human liver tissues. Lauschke et al. [15] performed studies in a model of isolated Wistar murine livers (non-heart-beating donors). Livers were stored at the University of Wisconsin (UW) fluid modified with SOD (600 U/mL) or taurine (0.5 mg/mL) at 4 °C for 24 h. An analysis of the collected perfusates showed that the addition of the antioxidant reduced enzyme release (LDH_SOD_ = 16.3 ± 4.7 U/L/g; LDH_TAURINE_ = 19.5 ± 7.3 U/L/g; LDH_CONTROL_ = 66.9 ± 17.4 U/L/g; GLDH_SOD_ = 498 ± 114 U/L/g; GLDH_TAURINE_ = 362 ± 108 U/L/g; GLDH_CONTROL_ = 924 ± 373 U/L/g), lipid peroxidation, and portal vascular resistance. Increased bile production was also observed. Taurine and SOD showed similar protective effects. Minor et al. [16] stored Wistar rat livers in UW fluid with or without TAU, then washed them with Ringer’s fluid and perfused them with Krebs–Henseleit buffer. The authors found that the addition of the antioxidant improved transhepatic flow. This could be explained by less tissue edema, which suggests an osmoprotective effect of TAU. In addition, they observed greater liver viability after reperfusion, which may be due to the involvement of TAU in modulating cellular calcium homeostasis.

### 3.2. Non-Enzymatic Antioxidants

#### 3.2.1. Low-Molecular-Weight Antioxidant

Glutathione is the most important endogenous, non-enzymatic systemic antioxidant that protects cells from oxidative stress. It is made up of three amino acids, L-glutamate, L-cysteine, and glycine, and is synthesized in the cytoplasm of cells. In biological cells, it is present in 99% of the reduced form (GSH) and 1% in the oxidized form (GSSG) and conjugates [67]. GSH is found in the cytoplasm, mitochondria, and cell nucleus, with concentrations ranging from 5 mM/L to 10 mM/L. In the endoplasmic reticulum, it is found at a concentration of 2 mM/L. Its antioxidant capacity is due to the presence of the thiol group -SH in the cysteine unit [68]. It participates in the repair of cell components damaged under oxidative stress, contributes to the restoration of reduced forms of thiol groups in proteins, reactivates lipid radicals, and reduces oxidized nitrogenous bases in DNA [69]. Studies conducted confirm its high efficacy as a component of the preservative fluid. Bardallo et al. [17], as expected, demonstrated a protective mechanism of action of glutathione in cells. GSH plays in the prevention of energy degradation in a model of isolated steatotic livers derived from homozygous Ob Zücker rats. Mitochondrial integrity was maintained. ATP production was maintained at optimal levels, succinate concentrations decreased, and expression of OXPHOS I and II (oxidative phosphorylation complexes), UCP2 (uncoupling protein 2), and PINK-1 (PTEN-induced kinase 1). Unfortunately, GSH is a critical component of preservative fluid. At pH = 7, it undergoes non-enzymatic autoxidation, which minimizes the antioxidant capacity of the solution and reduces its stability under storage conditions. Consequently, glutathione can act as an antioxidant or pro-oxidant, generating H_2_O_2_ [5,6,7]. Furthermore, the preparation of the GSH-containing liquid should be carried out under anaerobic conditions. Many authors have attempted to solve these problems by modifying fluid compositions with the addition of glutathione derivatives or its precursors. Of note is a series of studies by the team of Quintana et al. [18,19,20], who used S-Nitrosoglutathione (GSNO) to modify the composition of UW fluid. The authors used an isolated perfused rat liver model in their study. S-Nitrosoglutathione is a mediator of nitric oxide (NO) transmission. NO at low concentrations improves the hepatic microcirculatory status. GSNO at a concentration of 100 μM had an effect on maintaining the hepatic morphology and improved the flow rate of non-steatotic livers preserved during 48 h. Little vacuolation, fewer endothelial cells inside sinusoids, and good albumin distribution around central veins and middle zones were observed. Furthermore, the addition of GSNO reduced LDH activity, improved bile production, and reduced endothelial cell damage. The authors found no hepatoprotective effect of GSNO at concentrations of 50 µM, 250 µM, and 500 µM.

N-acetylcysteine (NAC) is a potent antioxidant that inhibits the activation of hepatic stellate cells (HSC). In the body, it is biotransformed to cysteine, which is a precursor of glutathione. In addition, it protects hepatocytes from apoptosis by affecting the JNK (c-Jun N-terminal kinases) signaling pathway and attenuates liver fibrosis [70,71]. NAC has been found to exert a renal vasodilatory effect by increasing nitric oxide production [72]. It improves liver function in patients with liver dysfunction and who are in septic shock [73]. Minor et al. [21] studied the effects of the University of Wisconsin fluid modified with the addition of superoxide dismutase (SOD) or N-acetylcysteine. In the study, they used livers from rats excised 60 min after cardiac arrest of the donor and stored under hypothermia (4 °C, 24 h). The authors confirmed that the addition of antioxidants prevented lipid peroxidation. NAC counteracted the phosphorylation of Iκb (an inhibitor of nuclear factor kappa). A similar trend was found by Srinivasan et al. [22] who conducted studies in an orthotopic liver transplantation model in rats. Storing livers in modified HTK fluid with 20 mM N-acetylcysteine increased antioxidant capacity, improved microcirculation, and reduced lipid peroxidation. The hepatoprotective effect of N-acetylcysteine in an animal model [5,6] was not confirmed in a subsequent human clinical trial. Aliakbarian et al. [55] added NAC to the UW fluid formulation and found that it had no effect on either reducing liver ischemia–reperfusion injury or short-term prognosis in transplant recipients (e.g., hospital stay, vascular complications, inotrope requirement, or in-hospital mortality). An interesting solution is the modification of UW fluid with the addition of S-Nitroso-N-Acetylcysteine (SNAC). SNAC is a synthetic compound that acts as a NO donor [8]. Scherer de Fraga et al. [23] conducted studies in an isolated rat liver model and found that SNAC solution presented a greater capacity to maintain hepatocyte integrity during cold preservation. The activity of the released AST enzyme was lower in the SNAC + UW group (823 U/L) vs. UW (959 U/L). SNAC (as an NO donor) could promote intrahepatic vasodilation and diminish the inflammatory process by decreasing the adhesion of platelets and leukocytes.

Deferoxamine (DFX) is a chelator that combines with iron in a ratio of 1:1. Iron ions catalyze reactions to form a hydroxyl radical with a very high oxidizing potential (Haber–Weiss reaction). Without the iron ions, the reaction rate would be too low for the reaction to be pathophysiologically relevant. The resulting hydroxyl radical promotes lipid peroxidation and reacts with proteins and mitochondrial DNA [74,75]. Kerkweg et al. [24] found that deferoxamine protects rat liver cells from lipid peroxidation and apoptosis. Increased concentrations of chelated iron reduce necrosis and apoptosis of hepatocytes during storage in modified UW + DFX fluid by simple hypothermia. Jain et al. [25] studied the efficacy of deferoxamine in a system with N-acetylcysteine and Trolox C using UW fluid. The proposed antioxidants had a protective effect on the structure of the liver stored in modified hypothermic machine perfusion (HMP) fluid. Decreased release of LDH and ALT enzymes, increased bile production, and ATP recovery were observed. Cell swelling decreased, suggesting improved liver microcirculation and mitochondrial function. Other authors [26] noted that DFX (added to UW fluid) inhibited oxidative stress in isolated hepatocytes. However, no similar antioxidant effect was observed in the perfused liver. This is probably due to the difference in the distribution of DFX in hepatocytes compared to the whole liver or its limited diffusion into cells during low-temperature storage of the graft.

Few studies on the efficacy of antioxidants as a component of preservative fluid have been conducted with HTK (Custodiol), which is recommended for all organs. This is presumably due to the presence of buffering histidine in the fluid formulation, which is toxic via increased intracellular chelate iron [27]. Studies available in the literature indicate that the traditional hydrophilic iron chelator, i.e., deferoxamine (DFX), and the new lipophilic hydroxamic acid derivative LK-614, which is membrane permeable, can be added to HTK fluid [27,28]. Wu et al. [27] investigated the efficacy of LK-614 in an isolated perfused rat liver model. Grafts were stored in cold HTK, mHTK fluid (chloride ion content reduced in fluid composition: 0.04 mM/L), or mHTK+ LK-614, respectively, for 24 h, and then reperfused for 60 min at T = 37 °C with Krebs–Henseleit buffer. LDH activity was lower in perfusates of the modified fluid. The bile produced in livers perfused with mHTK+ LK-614 fluid was higher. Hepatic microcirculation was also improved in this group of livers. In another study, Stegemann et al. [28] used two iron chelators, i.e., deferoxamine and LK-614, to modify the HTK fluid. Isolated rat livers were stored using the hypothermic machine perfusion (HMP) technique. The addition of DFX at a concentration of 25 µM and LK-614 at a concentration of 2.5 µM improved liver metabolic activity, significantly decreased cleavage of caspase 9, and abrogated positive signs of cellular apoptosis. The activity of released enzymes was significantly lower (ALT_HTK_ = 27.3 U/L vs. ALT_HTK+DFX+ LK-614_ = 0.6 U/L; LDH_HTK_ = 553 U/L vs. LDH_HTK+DFX+ LK-614_ = 139 U/L).

#### 3.2.2. Mitochondria-Targeted Antioxidants

SkQ1 is a synthetic antioxidant that neutralizes mtROS (mitochondrial reactive oxygen species). It also reduces TNF-α (tumor necrosis factor-alpha) induced endothelial damage and modulates angiogenesis [76]. Cherkashina et al. [29] studied the efficacy of this antioxidant in a hypothermic storage model of isolated rat liver. Grafts were placed for 24 h in fluid modified with the addition of 1 µM SkQ1, after which they were reperfused for 60 min (T = 37 °C). It was observed that the amount of ROS produced decreased. The activity of antioxidant enzymes (catalase, GSH peroxidase, GSH reductase, and glucose-6-phosphate dehydrogenase) decreased. The efficiency of hepatic energy processes was improved, increasing the respiratory control index of mitochondria and ATP levels. Increased bile flow during reperfusion was also found, confirming the improved secretory function of the organ. The authors highlighted that mitochondria-targeted antioxidants may exhibit cytoprotective effects.

Idebenone (QSA-10) is a short-chain benzoquinone, a synthetic analog of coenzyme Q-10, and has been found to be able to transfer electrons directly to complex III of the mitochondrial electron transport chain, restoring ATP production in the cell [77]. Wieland et al. [30] studied the efficacy of QSA-10 in a rat liver microsomal model incubated in a modified UW or HTK preservation solution. The antioxidant prevented reperfusion damage and showed hepatoprotective effects. Idebenone showed the ability to protect against liver lipid peroxidation and protein damage. A significant reduction in cytochrome P450 3A (CYP3A) levels was also observed. QSA-10 at a concentration of 20 µM/L provided a protective effect against protein damage as a component of both HTK and UW fluid. The addition of 0.1 µM/L QSA-10 to HTK fluid provided high liver protection against lipid peroxidation.

Phosphoenolpyruvate (PEP) is involved in glycolysis, where it is converted to pyruvate by pyruvate kinase in its final step. One molecule of PEP yields one molecule of ATP. It has been suggested that PEP may improve hepatic energy metabolism during ischemia [78]. Kondo et al. [31] studied the effect of PEP on oxidative damage to the graft in an ex vivo mouse liver cold preservation model, using phosphate-buffered saline (PBS) and UW fluids. Their study confirmed that PEP as a component of the preservation fluid showed the potential to scavenge reactive oxygen species, inhibit the increase in aminotransferase and lactate dehydrogenase activity, and attenuate ATP depletion. Storing livers in PBS + 100 mM PEP influenced a significant decrease in ALT, AST, and LDH activity, which was not observed in the UW + 100 mM PEP group. Analyzing oxidative stress parameters, there were no differences between the UW and UW + 100 mM PEP groups, while the efficacy of PBS + 100 mM PEP fluid in inhibiting the decrease in GSH concentration in graphite was confirmed. In contrast, ATP content was significantly higher in the UW + 100 mM PEP group compared to the PBS group. The authors noted that PEP showed a synergistic effect with the other components of the UW fluid only with regard to energy replenishment during the cold storage period of the liver.

α-Lipoic acid (ALA) is a cofactor of pyruvate dehydrogenase and α-ketoglutarate complexes and is involved in mitochondrial bioenergetic reactions. It has antioxidant and anti-inflammatory properties. It removes reactive oxygen species and has a positive effect on the state of the vascular endothelium. It shows the ability to regenerate other antioxidants: vitamins C and E, coenzyme Q10, and glutathione [79,80,81]. Aghdaie et al. [56] studied the efficacy of UW + α-lipoic acid and UW + ursodeoxycholic acid fluids in maintaining the vital functions of isolated human hepatocytes. Ursodeoxycholic acid (UDCA) belongs to the steroidal bile acids. It exhibits antioxidant and anti-inflammatory effects. According to many authors, it shows a hepatoprotective effect, while its toxic effects have also been demonstrated. This is probably due to the small difference between the recommended dose of UDCA (13 mg/kg/day) and the toxic dose (28 mg/kg/day). It can convert to lithocholic acid, which induces DNA strand breaks [82]. In a study [56], the authors found that both ALA and UDCA added to UW fluid did not increase the number of viable hepatocytes. The preservation of vital cell function was assessed by testing the ability of hepatocytes to synthesize urea. The mean number of viable hepatocytes stored in hypothermia (4 °C) in UW fluid was 66.3 ± 1.8% (urea concentration 9.21 ± 1.14 mg/dL), in UW fluid + α-lipoic acid it was 42.65 ± 2.19% (urea concentration 8.87 ± 0.62 mg/dL), and in UW fluid + ursodeoxycholic acid, it was 43.73 ± 2.55% (urea concentration 8.44 ± 0.91 mg/dL).

Horváth et al. [83] suggest that low temperature has a beneficial effect on mitochondrial metabolism, but that its optimal effect disappears above 12 h of SCS liver storage. The authors noted that the use of HMP and SNMP techniques ensures adequate mitochondrial function.

#### 3.2.3. Polyphenols

Curcumin, also known as long oyster, is a perennial herb in the ginger family. The compound is a diferuloylmethane and belongs to the hydrophobic polyphenol group of curcuminoids. It has been suggested that turmeric exhibits pleiotropic effects. Its antioxidant, anti-inflammatory, chemopreventive, anticancer, immunosuppressive, and hepatoprotective functions have been confirmed [84,85]. Studies in an animal model of nonalcoholic fatty liver disease (NAFLD) have confirmed its anti-steatotic and anti-fibrotic properties [86,87]. It can prevent the expression of ICAM-1 (intercellular adhesion molecule 1) and E-selectin in the pro-inflammatory vascular fluid that accumulates in the liver during clinical transplantation [88]. Ischemia-induced stress causes an increase in the expression of Hsp proteins. Turmeric has been found to be a potent stimulator of the heat shock proteins Hsp27, Hsp70, and alpha B crystalline, which are among the so-called ‘chaperone proteins’ [89]. In addition, it shows the ability to induce HO-1 (heme oxygenase-1), which protects cells from oxidative stress [90]. Chen et al. [33] found that the use of turmeric at a concentration of 100 µM as a component of UW and Euro-Collins (EC) preservative fluid improved the biochemical parameters of Sprague-Dawley rat livers. Curcumin increased portal vein flow velocity and bile production (bile output: EC_24h_ = 17 µLg min vs. EC + curcumin_24h_ = 43 µL/g min; UW_48h_ = 10.2 µL/g min vs. UW + curcumin_48h_ = 17.1µL/g min). Liver enzyme release into the perfusate was lower in the EC + curcumin group (AST: EC_24h_ = 11.4 g dry weight vs. EC + curcumin_24h_ = 5.7 g dry weight; UW_48h_ = 9.2 g dry weight vs. UW + curcumin_48h_ = 10.8 g dry weight; ALT: EC_24h_ = 13.2 g dry weight vs. EC + curcumin_24h_ = 6.2 g dry weight; UW_48h_ = 5.6 g dry weight vs. UW + curcumin_48h_ = 6.5 g dry weight). The authors concluded that curcumin increased the efficacy of preservative fluids in maintaining the vital functions of the isolated rat liver. McNally et al. [34] confirmed that UW fluid supplementation with curcumin protects human hepatocytes during cold preservation and warm reperfusion. This confirms its cytoprotective effect resulting from the induction of heme oxygenase-1. The protective role of curcumin in an ex vivo model of liver preservation was also confirmed by Johnston’s team [32].

Quercetin (QE) occurs naturally in plants. It has ‘GRAS’ status, indicating its high safety. It exhibits antioxidant, antimicrobial, anti-aggregative, anti-inflammatory, hypoglycemic, antiviral, anticancer, and liver-protective effects. It reduces oxidative stress and protects cells from damage. It inhibits the production of cytokines and inflammatory enzymes [91]. Kato et al. [35] studied the effect of quercetin added to UW fluid on the storage efficiency of isolated hepatocytes and the whole liver in rats. Grafts were stored in the fluid for 24 h at 4 °C. QE fluid supplementation at 0.33 µM/L and 33.1 µM/L improved cell viability to 28.3% and 23.7%, respectively. A decrease in ALT activity was observed in perfusate samples collected after liver lavage. ALT levels in UW fluid perfusates were 325 ± 110 IU/L, and in UW + QE fluid perfusates it was 80 ± 66 IU/L. In addition, sucrose (0.1 M/L) enhanced the effect of quercetin (ALT_UW+QE+suc_ = 28 ± 25 IU/L). The authors concluded that QE at 33.1 μmol/L showed efficacy in a rat model of liver undergoing cold preservation and orthotopic liver transplantation. It should be emphasized that the study was conducted under in vitro conditions. Another study was performed at the same research center on the efficacy of QE [57], which was added to UW fluid at a dose of 33.1 μM (in the presence of sucrose 0.1 M) using an isolated pig liver model. There was a significant static decrease in ALT, AST, and LDH enzyme activities in the group of grafts washed with UW + QE + Suc fluid. This solution prevented tissue edema. Histopathological examination also showed better results in terms of sinusoidal congestion and hepatocyte cytoplasmic vacuolization. The authors speculate that QE has a protective effect by suppressing oxidative stress. Given that quercitin shows poor bioavailability under in vivo conditions, further research is necessary.

Silibinin (SB) is a natural polyphenol, the most active component of the silymarin found in the fruit of the spotted thistle. It increases the activity of the enzymes dismutase and peroxidase, as well as glutathione concentration and glutathione peroxidase activity, thereby protecting liver cells from the oxidative effects of free oxygen radicals. It also regulates aldehyde oxidase activity in mitochondria and prevents mitochondrial dysfunction. It stabilizes cell membranes and inhibits prostaglandin synthesis associated with lipid peroxidation. It has anticancer, cholagogic, cholepoietic, anti-inflammatory, and potent detoxifying effects [92,93,94]. In a study conducted in an isolated rat liver model, the Ligeret team confirmed that silibinin shows potential in protecting hepatocytes [36]. UW fluid was used to assess the efficacy of SB. Livers were preserved in the modified fluid for 24 h at T = 4 °C. This was followed by reperfusion for 1 h at T = 37 °C. Silibinin at a dose of 100 µM increased mitochondrial ATP and RCR (respiratory control ratio) by 39% and 16%, respectively. A decrease in oxidative stress to values corresponding to control grafts (not preserved and not perfused) was also observed.

Magnolol (MAG) is a biphenol isolated in magnolia bark. It exhibits antioxidant, anticoagulant, anti-inflammatory, anticancer, neuroprotective, and antidiabetic properties. It protects mitochondria in a rat heart model against lipid peroxidation. Its antioxidant activity is due to its ability to scavenge oxygen free radicals and increase the activity of antioxidant enzymes [95,96]. Chiu et al. [37] stored isolated rat livers in UW + MAG or Ringer’s lactate + MAG fluids (the dose of magnolol was 10^−6^ M/L i.e., 2.66 mg/mL). This study showed that MAG was, respectively, 470 times more effective vs. α-tocopherol (α-T) in inhibiting oxygen consumption and 340 times more effective vs. α-T in inhibiting the formation of MDA (malondialdehyde) in mitochondria. In contrast, it had no significant effect on AST and ALT activity, which were determined in perfusate samples taken after 48 h and 96 h of graft storage in Ringer’s lactate + MAG. The authors conclude that magnolol inhibits lipid peroxidation in rat liver mitochondria.

#### 3.2.4. Bioactive Metabolites from Marine Algae

Phycocyanin (Pc) is a blue pigment found in spirulina, belonging to a class of compounds called phycobiliproteins. It has antioxidant, anti-inflammatory, antimicrobial, anticancer, and anti-neurodegenerative properties. It shows the ability to scavenge oxygen-free radicals and protect cells from oxidative damage [97]. The protective effect of phycocyanins on the liver was assessed by Gdara et al. [38] using an ex vivo model of isolated perfused rat liver. Livers after collection were stored for 12 and 24 h in fluids, respectively: KH (Krebs–Henseleit) or KH + Pc, cooled to 4 °C. Phycocyanin had a normalizing effect on the activity of aminotransferase (ALT, AST), phosphatase (ALP), lipid peroxidation (MDA), and the activity of antioxidant enzymes (glutathione-S-transferase (GST), glutathione peroxidase (GPx)). An increase in thiol groups in hepatic tissues was also observed. According to the authors, a Pc dose of 0.2 mg ml^−1^g^−1^ was optimal for renal protection. Phycocyanin at a concentration of 0.1 mg ml^−1^g^−1^ only decreased ALP and GST activity after 24 h of graft storage.

Fucoidan (FUC) is a sulfated polysaccharide extracted from brown algae. It has antioxidant, antiproliferative, and antiangiogenic properties. It also exhibits anticancer, immunostimulant, antioxidant, anti-inflammatory, analgesic, antithrombotic, antimicrobial, and antiviral effects [98]. Studies have confirmed the protective effect of FUC on the liver during ischemia. It contributes to reducing the developing inflammation (lowering TNF-ɑ, IL-1β, and CRP levels) and cell infiltration in inflamed areas. It is involved in the inhibition of hepatic expression of IL-6 (Interleukin-6) and PNPLA3 (Patatin-like phospholipase domain-containing protein 3) [99,100]. Slim et al. [39] assessed the effects of fucoidan on the vital functions of Wistar rat livers stored in a modified IGL-1 solution (graft preservation in cold fluid/4 °C for 24 h, reperfusion for 2 h at 37 °C, FUC dose: 100 mg/L). There was a decrease in the activity of aminotransferases (ALT, AST) and an increase in the phosphorylation of AMPK, AKT protein kinase, and GSK3-β. A reduction in cell apoptosis (caspase 3), an improvement in mitochondrial function, and a decrease in oxidative stress markers were observed.

#### 3.2.5. Vitamins and Vitamin-like Substances

α-Tocopherol (α-TCP) is a form of vitamin E with antioxidant, anti-inflammatory, and metabolic effects. It protects against the action of lipid-superoxide radicals. It exhibits the ability to block lipoxygenase activity. It influences the maintenance of the normal structure of membrane lipids. α-TCP prevents oxidative stress induced by TNF-α. It inhibits the formation of pro-inflammatory molecules, including IL-β. It exhibits a non-oxidative renal effect by stimulating activity on diacylglycerol kinase [101]. Bae et al. [40] added α-tocopherol to Vasosol fluid and conducted analyses of its efficacy in a rat model after cardiac death. Livers were stored for 8 h at 4 °C in UW fluid, then perfused using the hypothermic machine perfusion (HMP) technique with KPS-1, Vasosol, and Vasosol + α-TCP fluids. The authors confirmed that α-tocopherol reduced alanine aminotransferase (ALT) activity in the reperfusates. In addition, low levels of inflammatory cytokines (IL-6, TNF-α, MCP-1) were observed in the post-reperfusion biopsy, but the levels were not statistically significant. The levels of apoptosis markers (caspase 3 and 7) in the circulation also decreased, which was due to a reduction of the levels of Cytochrome C mRNA. α-Tocopherol enhances HMP efficacy with Vasosol.

Carnitine (CAR, β-hydroxy-γ-N-trimethylaminobutyric acid) is a vitamin-like substance. It belongs to naturally occurring hydrophilic amino acid derivatives. It is involved in the transport of activated long-chain fatty acids to β-oxidation sites in the mitochondria, resulting in the formation of ATP. It is involved in the regulation of acyl-CoA and the regeneration of free CoA in the mitochondrion and cytosol. In its role as an antioxidant, it prevents the accumulation of lipid peroxidation end products. It exhibits anti-apoptotic, anti-inflammatory, stabilizing biological membranes, and anti-fibrotic effects. It also plays an important role in modulating ketogenesis and glucogenesis [102,103]. Coskun et al. [42] evaluated the protective effects of L-carnitine on the vital functions of non-fatty liver (derived from a Wistar Albino rat) stored in UW or UW + L-CAR fluid for 48 h. It was observed that L-carnitine influenced a decrease in ALT (*p* < 0.05) and ACP (not statistically significant) activities in the collected perfusates and the levels of lipid peroxidation products (not statistically significant) in the analyzed tissues (UW: ALT = 268 IU/L; ACP = 2 IU/L; MDA = 5.0 nM/mg vs. UW + L-CAR: ALT = 171 IU/L; ACP = 1.9 IU/L; MDA = 4.1 nM/mg). In contrast, it had no significant effect on liver central vein dilatation, portal vein dilatation, sinusoidal dilatation, inflammation, autolysis, congestion, and edema. According to the authors, L-CAR can be an effective component of preservative fluid for flushing both fatty liver and non-fatty liver. The prophylactic effect of L-carnitine was also observed by the Tolba team [41]. In their study, L-CAR as a component of HTK fluid had a significant effect on reducing enzyme leakage from the steatotic livers of Wistar rats. ALT activity determined in post-reperfusion samples decreased by 70%, while GLDH activity decreased by 92%. Malondialdehyde (MDA) levels determined in the perfusate also decreased by 50% in the HTK + L-CAR group. In addition, the antioxidant prevented a decrease in hepatic energy metabolism. Oxygen consumption at the end of reperfusion was 28% higher in the group of grafts flushed with modified HTK fluid. Ultrastructural analysis showed no significant changes in the endoplasmic reticulum or mitochondria. The authors suggest that carnitine may act as a metabolic component in the composition of the preservation fluid, increasing liver viability after transplantation.

#### 3.2.6. Drugs

Carvedilol (CVD) belongs to the third generation of β-blockers. It is mainly used in the treatment of spontaneous hypertension and ischemic heart disease. It has been found to exhibit antioxidant, anti-inflammatory, anticoagulation, antiproliferative, cardioprotective, and neuroprotective effects. It influences endogenous nitric oxide (NO) production and improves vascular endothelial function. It blocks (depending on the concentration used) the production of oxygen free radicals in aqueous and lipid environments. It inhibits lipid peroxidation in the vascular endothelium and lipoprotein oxidation. Its antioxidant activity is suggested to be 10 times more potent than vitamin E [104,105,106]. Ben et al. [43] studied the effect of UW + CVD fluid on the vital functions of steatotic and non-steatotic livers stored using the simple hypothermia technique (SCS, 24 h) in an isolated rat liver model. Ex vivo perfusion was conducted at T = 37 °C for 2 h. Carvedilol reduced ALT and AST activity in perfusates taken from non-steatotic and steatotic livers. Bile production and hepatic clearance of sulfobromophthalein (BSP) improved. The activity of the mitochondrial damage marker GLDH (glutamate dehydrogenase) decreased in both liver types. The authors conclude that CVD via AMPK (adenosine monophosphate-activated protein kinase) increases NO production. In addition, it influences the preservation of higher ATP and adenine nucleotide content and reduces oxidative stress, thereby protecting the grafts from ischemia–reperfusion damage.

Trimetazidine (TMZ) is an antianginal and cardioprotective drug. By reducing β-oxidation of fatty acids at the level of 3-ketoacyl-co-enzyme A (CoA) thiolase (3-KAT), it secondarily enhances glucose oxidation, leading to an increase in cellular energy reserves when ischemia occurs. Reduces the excessive release of ROS and improves mitochondrial function. It influences the proper functioning of the sodium–potassium pump and cell homeostasis [107]. Ben et al. [44] studied the efficacy of TMZ in an isolated Zücker rat liver model. Steatotic and non-steatotic livers were stored for 24 h in UW and UW + TMZ fluids. Trimetazidine reduced transaminase activity (ALT and AST) and improved hepatic morphology in both liver types. The concentration of malondialdehyde (MDA), an indicator of the lipid peroxidation process, also decreased. Better bile production was observed. In a later report, the same authors [45] completed the study by adding aminoimidazole-4-carboxamide ribonucleoside (AICAR). AICAR is an adenosine monophosphate-activated protein kinase (AMPK) activator, which induces NO synthesis and protects against ischemia–reperfusion changes. The authors found that the separate addition of TMZ at a concentration of 10^−6^ M/L and AICAR at a concentration of 20 µM/L and 40 µM/L, respectively, to UW fluid-protected non-steatotic and steatotic livers during the ischemic period, and their efficacy remained similar. Both substances showed a similar protective effect against mitochondrial damage and ATP depletion. In contrast, the combination of TMZ and AICAR in UW fluid produced results similar to those obtained for UW + TMZ and UW + AICAR. The authors explain this by TMZ’s ability to activate AMPK, which increases the level of NO produced mainly from cNOS (constitutive nitric oxide synthase). They do not recommend modifying UW fluid with the addition of TMZ and AICAR simultaneously.

A series of studies on the modification of preservative fluid with the addition of trimetazidine was also carried out by Zaouali’s team [46,47,48,49]. They used a model of isolated steatotic and non-fatty rat livers. Grafts were stored in IGL-1 or IGL-1 fluid with TMZ at a concentration of 10^−6^ M/L for a period of 24 h at T = 4 °C, then subjected to 2 h of normothermic reperfusion. They found that TMZ induced NO and eNOS activation and prevented HIF-1α (hypoxia-inducible factor 1) degradation during reperfusion of grafts with the steatotic disease. ALT and AST release decreased after 2 h of reperfusion in both liver types. TMZ also had a beneficial effect on bile production and vascular resistance. HO-1 (heme oxygenase-1) expression increased, which conditioned better cytoprotection of grafts against ischemia–reperfusion injury [46]. The same authors in a subsequent study [47] evaluated the efficacy of melatonin and trimetazidine added to IGL-1 or UW fluid based on a previously developed experimental model. Melatonin is a hormone with antioxidant, anti-inflammatory, and anti-apoptotic effects. The authors found that the vital functions of steatotic livers were better preserved after preservation in the modified fluid. IGL-1 + TMZ + MEL influenced a significant decrease in GRP78 (glucose-regulated protein 78), pPERK (protein kinase R-like endoplasmic reticulum kinase), and CHOP (C/EBP homologous protein) levels. In addition, it enhanced AMPK-induced autophagy in the liver. A later study by Zaouali et al. [48] also confirmed that the inclusion of trimetazidine in the IGL-1 fluid formulation inhibited GSK3β (glycogen synthase kinase-3β) and VDAC (voltage-dependent anion channel), affecting the reduction of ER (endoplasmic reticulum) stress and preventing cell death during orthotopic liver transplantation. The modified fluid increased SIRT1 (sirtuin 1) levels, induced HIF-1α, and reduced HMGB1 (High-mobility Group Box 1 protein), thereby promoting autophagy in steatotic livers and increasing graft tolerance to ischemia–reperfusion injury [49]. Increasing SIRT1 levels inhibited the activity of rapamycin (mTOR is involved in T-cell activation and proliferation) through activation of HSP70 (heat shock protein 70) and AMPK [50].

Pentoxifylline (PTX) is a nonspecific phosphodiesterase inhibitor. It is used in the treatment of peripheral vascular disease. It improves the rheological properties of blood and minimizes the ability of erythrocytes to adhere and aggregate. It exhibits anti-inflammatory, immunoregulatory, anticoagulant, and antiproteinuric effects. There are data indicating its renoprotective effect in diabetic kidney disease [108]. Kozaki et al. [51] investigated the potential of PTX as a component of UW fluid in an isolated rat liver model. The organs were stored in UW fluid and UW + PTX for 4 or 24 h. Orthotopic graft transplantation was then performed. The authors showed that Kupffer cells produced significantly less TNF-α and O^2−^. Also, a study by Arnault et al. [52] in a 24 h fatty liver storage model gave satisfactory results. PTX counteracted the increase in vascular resistance. The activity of the released enzymes was comparable to that of a normal liver and lower compared to the control group of fatty livers (AST_UW_ = 42 IU/L/g of liver vs. AST_UW+PTX_ = 19 IU/L/g of the liver; ALT_UW_ = 32 IU/L/g of liver vs. ALT_UW+PTX_ = 11 IU/L/g of the liver; LDH_UW_ = 1 207 IU/L/g of liver vs. LDH_UW+PTX_ = 402 IU/L/g of the liver). The authors conclude that pentoxifylline improves microcirculation in the liver, which influences the preservation of parenchymal cell integrity. The addition of PTX to the preservation fluid extends the safe cold storage time limit of livers from 12 h to 16 h, as reported in a study by Qing et al. [58]. The authors investigated the efficacy of PTX in a simple porcine orthotopic liver transplantation model. Grafts were subjected to 20 min of warm ischemia, followed by 12 h, 16 h, and 20 h of preservation in UW and UW + PTX fluid. Fluid supplementation with pentoxifylline significantly reduced ALT and AST activities in recipients’ artery blood. The levels of MDA and TNF-alpha in grafted liver tissue, and resistance of portal vein and hepatic artery also decreased. According to the authors, the hepatic protective effect of PTX is due to its antioxidant activity, inhibition of lipid peroxidation, improvement of microcirculation, and energy metabolism of the graft.

M101 is an extracellular hemoglobin obtained from the Arenicola marina. It has been confirmed that one molecule of M101 can bind up to 156 oxygen molecules, while human hemoglobin can attach up to four molecules. Studies conducted in recent years confirm that it is an effective O_2_ carrier and exhibits antioxidant, anti-inflammatory, and antimicrobial activities. It is non-immunogenic and shows no toxicity. It has been confirmed in preclinical studies to improve cardiac, liver, kidney, pancreas, and lung function during transplantation [109]. One clinical trial was performed in a renal model (Clinical Trial Registry No. NCT 02652520) in which M101 at a dose of 1 g/L was added to UW fluid. The use of the innovative oxygen carrier was shown to be safe. Its beneficial effect on ischemia–reperfusion injury was confirmed [110]. Studies on the protective effects of M101 on the liver in the peri-transplantation period have been conducted by two scientific teams [53,59]. Alix et al. [59] evaluated the effect of adding M101 to UW fluid on the quality of pig livers in an orthotopic allotransplantation (OLT) model. Livers were stored by simple hypothermia (SCS) using UW and UW + M101 fluids and by hypothermic oxygen perfusion (HOPE). It was found that the use of the HOPE technique and SCS + UW with M101 improved graft function parameters, i.e., mitochondrial function, ATP synthesis, architecture, and antioxidant capacity of hepatocytes. The ATP concentration after 9 h of preservation was UWscs + M101 = 2.4 µM/mg protein vs. HOPE = 6.4 µM/mg protein. The use of both preservation techniques resulted in a decrease in inflammatory mediators and oxygen-free radical production. M101 affected oxygenates of liver grafts during preservation. However, the authors emphasize that liver function was better preserved using the HOPE technique than SCS + UW with M101. The effective effect of M101 in the preservation of isolated steatotic rat liver was also confirmed by Asong-Fontem et al. [53]. Grafts were stored for 24 h in IGL-1 and IGL-1 + M101 fluids during SCS, and then subjected to 2 h of normothermic reperfusion. M101 decreased transaminase activity, GLDH (glutamate dehydrogenase), and lactate levels. A decrease in MDA levels, higher nitrite and nitrate production, and a decrease in the inflammatory mediator HMGB1 (High-mobility Group Box 1) were also observed. M101 attenuates damage induced by ischemia and reperfusion.

Analyzing the available literature, there is considerable scientific interest in optimizing preservation fluids with the addition of antioxidants. The results of the review confirm their therapeutic potential in protecting the liver before transplantation. Although most of the studies conducted support the hypothesis of hepatoprotective properties of antioxidants, it is uncertain whether the observed effects in vitro will be reflected in vivo. The vast majority of experimental studies have been carried out in rat, pig, or cell models in culture, using doses of antioxidants that have mostly not been optimized.

Only six preclinical and clinical studies were found in the available literature (Table 2). As mentioned for NAC [56], its hepatoprotective effect in an animal model has not been confirmed in humans. UW fluid modification did not affect the incidence of ischemia–reperfusion injury or short-term liver transplantation outcomes. The efficacy of α-Lipoic acid as a component of UW fluid was also not demonstrated [57]. This antioxidant did not improve the viability of human hepatocytes. Only the addition of rMnSOD to Celsior fluid protected human liver tissues collected from a deceased donor by counteracting oxidative stress [55].

## 4. Perfusion Methods Considerations 

Despite the rapid development of transplantation medicine, including liver storage techniques, the availability of grafts for transplantation is still a major problem. To increase the organ pool, advanced research is being conducted to improve the quality of marginal grafts from steatotic livers and those harvested from extended criteria donors (ECD). By using normothermic machine perfusion, the percentage of grafts eligible for transplantation can be increased. This method allows real-time liver and bile duct function assessment and can predict short-term transplantation outcomes [111]. It has been suggested that using NMP after the initial SCS period facilitates the regeneration of impaired cellular metabolic processes [112]. Assessment of graft function is based on analysis of selected parameters, including hemodynamic stability, bile production, and perfusate lactate clearance [113]. However, to date, there has been no standardization of diagnostic biomarkers to enable clinical evaluation of grafts [114]. This technique reduces macrovascular hepatic steatosis and potentially offers the possibility of storing the liver for up to 7–10 days [115,116].

Some authors indicate that it is advantageous to combine two liver storage techniques, e.g., SCS (at the stage of transporting the organ to the transplant center that has qualified the recipient) and NMP (to safely extend the preservation time of marginal organs or to perform the necessary perioperative procedures for demanding recipients). This is mainly due to challenges in managing the logistics of transporting NMP equipment (not all machines available on the market are portable), the need for trained personnel at the donor/recipient site, and the higher cost of the procedure [117]. Studies conducted using NMP after a period of static cold storage (SCS) confirm the high efficacy of combining the two techniques in high-risk liver transplantation. Laing et al. [118], based on a study in the UK, showed that 71% of organs originally unsuitable for transplantation could be transplanted using this method with 100% 90-day recipient/liver survival. Liu et al. [119], on the other hand, investigated the efficacy of liver storage with hypothermic oxygenated perfusion (HOP) and normothermic machine perfusion techniques. They concluded that the use of HOP-NMP was safe and provided potential benefits in preserving the function of grafts harvested from expanded criteria donors. Boteon et al. [120] suggest that a combined protocol of HOP and NMP alleviates oxidative stress, minimizes the risk of tissue inflammation, and improves metabolic recovery of marginal livers.

Critical to minimizing the effects of ischemic graft injury, especially in terms of protecting marginal grafts, is the development of machine perfusion and optimizing the composition of organ storage fluids. Data from the European Liver Transplant Registry (ELTR) indicate that the most commonly used fluids for liver transplantation are HTK, UW, and IGL-1 [121,122]. The use of HTK fluid has recently been restricted due to the concerns of the United Network for Organ Sharing (UNOS) [12]. It was observed that an increase in PNF (primary nonfunction) correlated with an increase in the proportion of livers flushed with HTK solution. Preston et al. [123] suggest an increased risk of deceased donor liver transplant failure resulting from the use of HTK fluid. Several reports of the beneficial properties of HTK fluid have been published. The lower concentration of K^+^ ions in its composition protects against hyperkalemic cardiac arrest upon reperfusion. Some of the authors suggest that it is more cost effective to use HTK fluid, whose consumption for graft washing is lower compared to UW fluid (422 5 mL vs. 5500 mL, *p* = 0.04) [124]. The lower viscosity of HTK fluid results in better microvascular perfusion [123]. Karakoyun et al. [14] confirmed the comparable efficacy of UW and HTK fluids in patient and graft survival. They observed a lower incidence of post-transplant biliary stenosis for grafts flushed with HTK fluid compared to UW fluid (13.5% vs. 22.7%, *p* = 0.013). IGL-1 is a relatively new fluid that was developed based on the composition of UW fluid. It is an extracellular fluid with a high concentration of sodium (120 mM/L) and a low concentration of potassium (25 mM/L), which helps to minimize the risk of cardiovascular complications. The inclusion of polyethylene glycol (PEG 35) in place of hydroxyethyl starch (HES) in the composition of IGL-1 fluid reduced its viscosity to 1.25 mm^2^/s [125]. Szilágyi et al. [126] found comparable efficacy of IGL-1 fluid with UW and HTK fluids in protecting deceased donor livers (DDL). They found no statistical difference in the incidence of PNF after the use of either fluid. More recently, experimental studies analyzing the efficacy of UW fluid as an alternative NMP perfusate in renal transplantation have been conducted [127].

Analyzing the data included in the review, it can be concluded that the function of the graft is influenced by the time and method of storage in the modified preservation fluid. The shorter the time, the better the function of the organ and, consequently, the more efficient the uptake of vital functions after transplantation. Work comparing the efficacy of SCS and MP techniques indicates that better organ preservation is achieved using dynamic liver preservation. The use of the HMP technique has a significant effect on lowering the activity/concentration of markers of liver function in grafts harvested from marginal donors. The addition of an antioxidant further improves the benefits of the HMP technique [26]. It is noteworthy that only 7% of the papers analyzed used extracorporeal mechanical perfusion in hypothermia to assess the efficacy of modified preservation fluid. Most of the information cited is from reports of studies conducted in animal models, most of them in rodents. None of the teams proposed to conduct parallel studies in a human and animal liver transplantation model. Only one study was conducted in a human clinical trial. Despite the promising results of antioxidant efficacy obtained in an animal model, the benefit of antioxidants in a clinical trial cannot be predicted.

## 5. Conclusions

An analysis of the literature indicates a beneficial effect of most antioxidants used in preservative fluid supplementation. However, it should be emphasized that most of the information cited comes from reports of studies carried out on animal models, most of them on rodents. With regard to the current state of knowledge, it is important to establish an effective and safe antioxidant dose (taking into account direct exposure to the organ). It is also crucial to develop the technology for the preparation of the fluid containing the readily oxidizable substance. Previous studies confirm that technological errors during fluid preparation may result in oxidation of the antioxidant and contamination of the preparation with reactive iron, which may consequently reduce the effectiveness and safety of the preservative fluid. Consideration should also be given to developing a carrier to facilitate the diffusion of the antioxidant into the cell (e.g., in the form of a liposome). In order to further our knowledge of the efficacy of antioxidants added to preservation fluids in protecting the liver prior to transplantation, studies should be continued with other animal models and other research protocols, taking into account, among other things, a dynamic organ preservation strategy. I also suggest modifying the composition of preservation fluids with the addition of antioxidants in combination with hormones with antioxidant properties (e.g., prolactin, melatonin), which could potentially intensify the hepatoprotective effect of the fluid.

## Figures and Tables

**Table 1:** Studies on the effectiveness of supplementing preservative fluids with antioxidants. Basic research.

Author, Year of Publication	Antioxidant	Species	Preservation Solution Modification /Cold Ischemia	Outcome Measures, (Intervention, I/Control, C)	Antioxidant Dose	Effects of Antioxidant
Enzymatic antioxidant						
Lauschke et al., 2003 [15]	Taurine SOD	Isolated perfused rat liver model	UW 24 h; 4 °C; SCS with VSOP	I1: UW + SOD I2: UW + TAU C: UW	SOD: 600 U/mL; Taurine: 0.5 mg/mL	↓ lipid peroxidation; ↓ vascular resistance; ↓ LDH, GLDH; ↑ bile production
Minor et al., 1995 [16]	Taurine	Wistar rats	UW 24 h; 4 °C; SCS	I: UW + TAU C1: UW	1 mM/L	improve hepatic circulation; enhance viability of the liver upon reperfusion
Non-enzymatic antioxidants						
Low-molecular-weight antioxidant						
Bardallo et al., 2022 [17]	PEG35, Glutathione	Zücker rats	IGL 24 h; 4 °C; SCS	I1: IGL + GSH I2: IGL + PEG35 + GSH C: IGL	PEG35: 1 g/L, 5 g/L GSH: 3 mM/L, 9 mM/L	IGL + PEG35 (5 g/L) + GSH (9 mM/L): maintained ATP production; ↓ succinate accumulation; ↑ expression of the OXPHOS complexes, UCP2, PINK-1, Nrf2, and HO-1; protected against lipid and protein oxidation; increased the GSH/GSSG ratio; ↓ inflammasome NLRP3 expression; protecting mitochondrial integrity
Quintana et al., 2001 [18]	S-Nitrosoglutathione	Isolated perfused rat liver model	UW 48 h; 4 °C; SCS	I: UW + GSNO C: UW	100 µM	the hepatic morphology was conserved showing little vacuolation; avoiding hepatic injury post cold preservation/reperfusion
Quintana et al., 2002 [19]	S-Nitrosoglutathione	Isolated perfused rat liver model	UW 48 h; 0 °C; SCS	I: UW + GSNO C: UW	50 µM, 100 µM, 250 µM, 500 µM	100 µM: prevented the ischemia/reperfusion injuries; ↓ LDH; improved bile production; partially reduced endothelial cell damage
Quintana et al., 2004 [20]	S-Nitrosoglutathione	Isolated perfused rat liver model	UW 48 h; 0 °C; SCS	I: UW + GSNO C: UW	500 μM 100 μM	500 μM: interstitial edema after normothermic reperfusion; 100 μM: damages on mast cells were avoided
Minor et al., 2000 [21]	N-Acetylcysteine, SOD	Isolated perfused rat liver model	UW 24 h; 4 °C; SCS VSOP	I1: UW + SOD I2: UW + NAC C: UW	SOD: 600 U/mL; NAC: 20 mM	prevented an increase in free radical-mediated lipid peroxidation; NAC counteracted the phosphorylation of Iκb
Srinivasan et al., 2014 [22]	N-Acetylcysteine	Lewis rat	HTK 1 h, 3 h, 24 h, 168 h; 5 °C; SCS	I: HTK + NAC C: HTK	20 mM	↓ PVP; ↓ ALT; improved microcirculation; diminished histologic graft damage; ↓ lipid peroxidation; ↑ total antioxidant capacity
Scherer de Fraga et al., 2010 [23]	S-Nitroso-N-Acetylcysteine	Wistar rats	UW 2 h, 4 h, 6 h; 4 °C; SCS	I: UW + SNAC C: UW	200 nM	↓ AST ↓ Liver injury
Kerkweg et al., 2002 [24]	Deferoxamine	Hepatocytes from male Wistar rats, rat liver endothelial cells	UW 24 h; 4 °C; SCS	I: UW + DFX C: UW	10 mM	↓ lipid peroxidation; ↓ apoptosis
Jain et al., 2008 [25]	N-Acetylcysteine, Trolox C, Deferoxamine	Isolated perfused rat liver model	UW 4 °C; HMP	I: UW + Gly + NAC + TRX-C + DFX C: UW	NAC: 5 mM TRX-C: 0.2 mM DFX: 0.25 mM	↓ LDH, ALT; ↑ bile production; improved mitochondrial function; improved liver microcirculation; intact hepatocytes
Vreugdenhil et al., 1997 [26]	Deferoxamine, Trolox C, Dithiothreitol	Hepatocytes from Sprague-Dawley rats; isolated perfused rat liver model	UW 24 h, 48 h; 4 °C; SCS	I1: UW + DFX I2: UW + TRX I3: UW + DTT C: UW	DFX: 2.5 mM, 5 mM, 10 mM TRX: 3 mM, 5 mM, 10 mM DTT: 5 mM, 10 mM, 20 mM	poor distribution of antioxidants in isolated rat liver; only DFX was effective when added to the UW: suppressed oxidative stress only in isolated hepatocytes stored in cold storage; ↓ LDH
Wu et al., 2009 [27]	LK 614	Isolated perfused rat liver model	HTK 24 h; 4 °C; SCS	I: HTK + LK 614 C: HTK	20 μM	↓ LDH during reperfusion; increased bile secretion; better preserved hepatic microcirculation
Stegemann et al., 2010 [28]	Deferoxamine, LK 614	Wistar rats	HTK 18 h; 4 °C; HMP	I: HTK-N + LK 614 + DFX C1: HTK C2:HTK-N	DFX: 25 μM LK 614: 2.5 μM, 7.5 μM	↓ ALT, LDH; DFX: 25μM and LK 614: 2.5μM improved metabolic activity, reduced cleavage of caspase 9 and apoptotic index
Mitochondria-targeted antioxidants						
Cherkashina et al., 2011 [29]	SkQ1	Rat	Sucrose–saline 24 h; 4 °C; SCS	I: Sucrose–saline + SkQ1 C: Sucrose–saline	1 μM	↓ hepatic injury and oxidative stress; ↑ liver and mitochondrial function
Wieland et al., 1995 [30]	Idebenone (QSA-10)	Rat liver microsomal model	UW, HTK 4 °C; SCS	I1: UW + QSA-10 I2: UW + Q-10 I3: HTK + QSA-10 I4: HTK + Q-10 C1: UW C2: HTK	QSA-10: 0.1 µM/L, 20 µM/L Q-10: 20 µM/L, 100 µM/L	protection against lipid peroxidation (HTK + QSA-10: 0.1 µM/L); prevented protein damage (HTK + QSA-10: 20 µM/L and UW + QSA-10: 20 µM/L); Q-10: 20 µM/ partial protection in UW; QSA-10 have the potential to increase the efficacy of organ preservation
Kondo et al., 2013 [31]	Phosphoenolpyruvate	Mouse liver (ex vivo)	UW, PBS 24 h, 48 h, 72 h, 4 °C; SCS	I1: UW + PEP I2: PBS + PEP C1: UW C2: PBS	PEP: 1 mM, 10 mM, 100 mM (PBS) PEP: 100 mM (UW)	↓ oxidative stress; attenuate ATP depletion; prevents increases in biochemical parameters
Polyphenols						
Johnston et al., 1999 [32]	Curcumin	Sprague-Dawley rat livers (ex vivo)	UW, EC 24 h; 4 °C; oxygenated perfusion	I1: EC+ CUR C1: EC C2: UW	100 μM	curcumin-enhanced EC solution was equivalent to the UW solution
Chen et al., 2006 [33]	Curcumin	Sprague-Dawley rat livers	UW, EC, PBS 24 h, 36 h, 48 h; 4 °C; SCS	I1: UW + CUR I2: EC+ CUR I3: PBS+ CUR C1: UW C2: EC C3: PBS	25–200 μM	curcumin at 100 μM concentration had the optimal preservation characteristics; ↑ portal flow rates and bile production; ↓ ALT, AST, LDH; improves the quality of organs
McNally et al., 2006 [34]	Curcumin	Human hepatocytes	UW 16 h, 24 h, 48 h, 72 h; 4 °C; SCS	I: UW + CUR C: UW	10 μM	induces HO-1; maximum protection cells between 16 and 24 h of CS
Kato et al., 2020 [35]	Quercetin	Rat (isolated hepatocytes and whole liver)	UW 24 h; 4 °C; SCS	I: UW + QE C: UW	QE: 0.33; 33.1 µM/L Suc: 0.1 M/L	optimal dose of QE: 33,1 µM/L ↓ ALT mild vascular degradation; improvement of histological changes
Ligeret et al., 2008 [36]	Silibinin	Isolated perfused rat liver model	UW 24 h; 4 °C; SCS	I: UW + SB C: UW	100 μM	↑ ATP, RCR ↓ oxidative stress
Chiu et al., 1999 [37]	Magnolol	Rat	UW, Ringer’s lactate 24 h, 48 h; 96 h, 4 °C; SCS	I1: UW + MAG I2: Ringer’s lactate + MAG C1: UW C2: Ringer’s lactate	10–6 M/L	↓ lipid peroxidation
Bioactive metabolites from marine algae						
Gdara et al., 2018 [38]	Phycocyanin	Rat	KH 12 h, 24 h; 4 °C; SCS	I: KH + Pc C:KH	0.1; 0.2 mg ml^−1^ g^−1^ of liver	optimal dose of Pc: 0.1 mg ml^−1^ g^−1^; ↓ ALT, AST, ALP; ↓ MDA; ↓ GST, GPx
Slim et al., 2020 [39]	Fucoidan	Isolated perfused rat liver model	IGL-1 24 h, 4 °C; SCS	I1: IGL-1 + FUC C1: IGL-1 C2: Ringer’s lactate	10 mg/L, 50 mg/L, 100 mg/L, 250 mg/L	optimal dose of FUC: 100 mg/L; ↓ ALT, AST; ↑ phosphorylation of AMPK, AKT protein kinase, and GSK-3β; reduction in apoptosis (caspase 3); reduction of mitochondrial damage; reduction oxidative stress; reduction of ER stress markers; ↓ ERK1/2 and p38 MAPKs phosphorylation
Vitamins and vitamin-like substances						
Bae et al., 2014 [40]	α-Tocopherol	Wistar rats after cardiac death	Vasosol 4 °C; HMP	I: Vasosol + α-TCP C1: Vasosol C2: KPS-1	5.4 × 10^−2^ mM	↓ ALT; ↓ inflammatory cytokines (IL-6, TNF-α, MCP-1); ↓ caspase 3/7 expression in the circulation
Tolba et al., 2003 [41]	L-carnitine	Isolated perfused rat liver model	HTK 24 h, 4 °C; SCS	I: HTK + L-CAR C: HTK	5 mM	↓ ALT, GLDH; improved the hepatic energy metabolism; preserved integrity of the mitochondria and the endoplasmic reticulum
Coskun et al., 2007 [42]	L-carnitine	Wistar Albino rat	UW 2 h, 24 h, 36 h, 48 h; 4 °C; SCS	I: UW + L-CAR C: UW	5 mM/L	↓ ALT, ACP; ↓ MDA
Drugs						
Ben et al., 2010 [43]	Carvedilol	Isolated Zücker rat liver (steatotic and non-steatotic)	UW 24 h; 4 °C; SCS	I: UW + CVD C: UW	10^−5^ M/L	reduced hepatic injury, and improved hepatic functionality in both liver types
Ben et al., 2006 [44]	Trimetazidine	Zücker rats (steatotic and non-steatotic)	UW 24 h; 4 °C; SCS	I: UW + TMZ C: UW	10^−6^ M/L	protects against mitochondrial damage; preserves more ATP; decreases oxidative stress higher bile production
Ben et al., 2007 [45]	Trimetazidine, aminoimidazole-4-carboxamide ribonucleoside	Zücker rats (steatotic and non-steatotic)	UW 24 h; 4 °C; SCS	I1: UW + TMZ I2: UW + AICAR C: UW	TMZ: 10^−8^ M/L 10^−6^ M/L, 10^−4^ M/L AICAR: 10 μM/L, 20 μM/L, 40 μM/L, 80 μM/L TMZ + AICAR: 10^−6^ M/L + 20 μM/L; 10^−6^ M/L + 40 μM/L;	↓ AST (TMZ) TMZ improved bile production; increase in cNOS via increasing AMPK; TMZ and AICAR protected, with a similar degree of effectiveness, against cold I/R injury in steatotic and non-steatotic livers; not necessary to combine AICAR and TMZ
Zaouali et al., 2010 [46]	Trimetazidine	Zücker rats	IGL-1 24 h; 4 °C; SCS	I: IGL-1 + TMZ C: IGL-1	10^−6^ M/L	↓ AST, ALT; higher bile production; increased NO production; HIF-1α accumulation; increased HO-1 expression
Zaouali et al., 2013 [47]	Trimetazidine melatonin	Zücker rats (steatotic)	UW, IGL-1 24 h, 4 °C; SCS	I2: IGL-1 + TMZ +MEL C1: IGL-1 C2: UW	TMZ: 10^−3^ µM MEL: 100 µM	↑ liver autophagy; ↓ GRP78, pPERK, and CHOP after reperfusion; improved steatotic liver preservation through AMPK activation; synergism of action MEL and TMZ
Zaouali et al., 2017 [48]	Trimetazidine	Sprague-Dawley rats	UW, IGL-1 8 h, 4 °C; SCS	I1: IGL-1 + TMZ C: IGL-1 C: UW	10^−6^ M/L	↓ ALT, GLDH, MDA; protects the mitochondria; inhibited of GSK3β and VDAC phosphorylation; reduced apoptosis; decreased ER stress
Zaouali et al., 2017 [49]	Trimetazidine	Homozygous obese and lean Zücker rats	IGL-1 24 h, 4 °C; SCS	I: IGL-1 + TMZ C: IGL-1	10^−5^ M/L	↓ ALT, AST (especially in steatotic livers); ↑ SIRT1 protein levels; ↓ HMGB1 protein level; ↓ TNF-α release; increasing the tolerance of steatotic liver graft against cold IRI
Pantazi et al., 2015 [50]	Trimetazidine	Rat orthotopic liver transplantation	IGL-1 8 h, 4 °C; SCS	I: IGL-1 + TMZ C: IGL-1	10^−6^ M/L	↓ ALT, GLDH; Reduction of oxidative stress; reduction of mitochondrial damage; enhanced SIRT1 protein expression
Kozaki et al., 1995 [51]	Pentoxifylline	Isolated perfused rat liver model	UW 4 h, 24 h; 0–4 °C; SCS	I: UW + PTX C: UW	25 mg PTX/kg body weight	the Kupffer cells produced significantly less O_2_^−^ and TNF-α
Arnault et al., 2003 [52]	Pentoxifylline	Isolated perfused Wistar rat liver model	UW 18 h, 24 h; 4 °C; SCS	I: UW + PTX C: UW	30 mM (during cold storage) 3 mM (at reperfusion);	improve microcirculation in the liver; decrease in vascular resistance at reperfusion of 18 h and 24 h; decreased number of foci of peliosis after an 18 h preservation; ↓ LDH, AST, ALT after 24 h cold ischemia time
Asong-Fontem et al., 2021 [53]	M101	Zücker rats	IGL-1 24 h, 4 °C, SCS 2 h, 37 °C, NR	I: IGL-1 + M101 C: IGL-1	1 g/L	↓ AST, ALT, Lactate, GLDH; ↓ MDA; higher production of NO2-NO3; less inflammation (HMGB1)

**Table 2:** Studies on the effectiveness of supplementing preservative fluids with antioxidants. Preclinical and clinical studies.

Author, Year of Publication	Antioxidant	Species	Preservation Solution Modification /Cold Ischemia	Outcome Measures, (Intervention, I/Control, C)	Antioxidant Dose	Effects of Antioxidant
Enzymatic antioxidant						
Hide et al., 2014 [54]	rMnSOD	human liver samples, LSEC, liver grafts from healthy and steatotic rats	Celsior 16 h, 4 °C; SCS	I: Celsior + rMnSOD C: no SCS	rMnSOD: 0,15 µM	↓ oxidative stress; ↑ NO;
Non-enzymatic antioxidants						
Low-molecular-weight antioxidant						
Aliakbarian et al., 2017 [55]	N-Acetylcysteine	Human	UW 4 °C; SCS	I: UW + NAC C: UW	2 g	addition of NAC does not decrease the rate of ischemia–reperfusion injury
Mitochondria-targeted antioxidants						
Aghdaie et al., 2019 [56]	α-Lipoic acid (ALA) ursodeoxycholic acid (UDCA)	Isolated human hepatocytes derived from livers of deceased donors	UW 24 h; 4 °C; SCS	I1: UW + α-Lipoic acid I2: UW + ursodeoxycholic acid C: UW	ALA: 5 mM/L UDCA: 5 mM/L	does not increase the number of viable hepatocytes
Polyphenols						
Otani et al., 2023 [57]	Quercetin	Isolated pig livers	UW 6 h, 4 °C; SCS	I: UW+ QE C:UW	QE: 33.1 µM/L Suc: 0.1 M/L	↓ ALT, AST, LDH; improvement of histological changes; prevent tissue edema
Drugs						
Qing et al., 2006 [58]	Pentoxifylline	Simple porcine orthotopic liver transplantation	UW 12 h, 16 h, 20 h; 4 °C; SCS	I: UW + PTX C: UW	1 g/L	↓ TNF-α, MDA; ↓ ALT, AST; ↑ ATP; 100% 1-week survival; improved microcirculation
Alix et al., 2020 [59]	M101	Pig allogeneic liver orthotopic transplantation	UW 9 h, 4 °C; SCS	I: UW + M101 C: UW	1 g/L	improved liver graft oxygenation during SCS; livers cold stored with UWSCS + M101 did not reach the oxygenation level achieved with machine perfusion

Abbreviations: ACP, acid phosphatase; AICAR, aminoimidazole-4-carboxamide ribonucleoside; ALA, α-Lipoic acid; ALT, alanine aminotransferase; AMI, amifostine; AMPK, AMP-activated protein kinase; AraA, adenine9-b-D-arabinofuranoside; AST, aspartate transferase; ATP, adenosine triphosphate; CHOP, C/EBP homologous protein; cNOS, constitutive nitric oxide synthase; CUR, curcumin; CVD, carvedilol; DFX, Deferoxamine; DTT, Dithiothreitol; EC, Euro-Collins solution; ER, endoplasmic reticulum; ERK1/2, extracellular signal-regulated protein kinase; FUC, Fucoidan; GLDH, glutamate dehydrogenase; Gly, glycine; GRP78, glucose-regulated protein 78; GSH, reduced glutathione; GSK-3β, glycogen synthase kinase 3 beta; GSNO, S-Nitrosoglutathione; GSSG, oxidized glutathione; HMGB1, High-mobility Group Box 1; HMP, hypothermic machine perfusion; HO-1, heme oxygenase 1; HOPE, hypothermic oxygen perfusion; HTK, histidine–tryptophan–ketoglutarate; IL-6, interleukin 6; Iκb, inhibitor of nuclear factor kappa; KH, Krebs–Henseleit; KPS-1, kidney machine perfusion solution; L-CAR, L-carnitine; LDH, lactate dehydrogenase; LSEC, liver sinusoidal endothelial cell; M101, Hemo2Life^®^; MAG, magnolol; MCP-1, monocyte chemotactic protein 1;MEL, melatonin; NAC, N-Acetylcysteine; NLRP3, pyrin domain-containing protein 3; NR, normothermic reperfusion; Nrf2, nuclear factor-erythroid 2-related factor 2; OXPHOS, oxidative phosphorylation complexes; p38 MAPK, mitogen-activated protein kinase; PBS, phosphate buffer saline; PEP, phosphoenolpyruvate; PINK1, PTEN-induced kinase 1; pPERK, protein kinase R-like endoplasmic reticulum kinase; PTX, Pentoxifylline; PVP, portal venous pressure; QE, quercetin; QSA-10, idebenone; RCR, respiratory control ratio; RL, Ringer Lactate; SB, silibinin; SCS, static cold storage; SIRT1, Sirtuin 1; p-p38, phosphor-p38; SkQ1, 10-(6′-plastoquinonyl)decyltriphenylphosphonium; SNAC, S-nitroso-N-acetylcysteine; Suc, sucrose; TAU, taurine; TMZ; trimetazidine; TNF-α, tumor necrosis factor α; TRX-C, Trolox-C; UCP2, uncoupling protein 2; UDCA, ursodeoxycholic acid; UW, University of Wisconsin solution; VDAC, voltage-dependent anion channel; VSOP, venous systemic oxygen persufflation; α-TCP, α-Tocopherol; ↑ increase ↓ decrease.

## Data Availability

The data presented in this study are available on request from the corresponding author.
